# Event-related potential response to auditory social stimuli, parent-reported social communicative deficits and autism risk in school-aged children with congenital visual impairment

**DOI:** 10.1016/j.dcn.2017.07.003

**Published:** 2017-07-19

**Authors:** Joe Bathelt, Naomi Dale, Michelle de Haan

**Affiliations:** a MRC Cognition & Brain Sciences Unit, University of Cambridge, Cambridge, United Kingdom; b Developmental Vision Clinic, Great Ormond Street Hospital for Children NHS Foundation Trust, United Kingdom; c Developmental Neurosciences, UCL Great Ormond Street Institute of Child Health, United Kingdom

**Keywords:** Social development, Childhood, Visual impairment, Autism spectrum disorder, Event-related potentials

## Abstract

Communication with visual signals, like facial expression, is important in early social development, but the question if these signals are necessary for typical social development remains to be addressed. The potential impact on social development of being born with no or very low levels of vision is therefore of high theoretical and clinical interest. The current study investigated event-related potential responses to basic social stimuli in a rare group of school-aged children with congenital visual disorders of the anterior visual system (globe of the eye, retina, anterior optic nerve). Early-latency event-related potential responses showed no difference between the VI and control group, suggesting similar initial auditory processing. However, the mean amplitude over central and right frontal channels between 280 and 320 ms was reduced in response to own-name stimuli, but not control stimuli, in children with VI suggesting differences in social processing. Children with VI also showed an increased rate of autistic-related behaviours, pragmatic language deficits, as well as peer relationship and emotional problems on standard parent questionnaires. These findings suggest that vision may be necessary for the typical development of social processing across modalities.

## 1 Introduction

The ability of adults to infer the mental state of others by interpreting fleeting contractions of facial muscles is an amazing feat of our species. Developmental studies show that infants are intuitively drawn to faces (Johnson et al., 1991, Johnson et al., 2000) and engage in reciprocal communication with their caregivers through facial expressions from just a few months of age (Leppänen and Nelson, 2008). Non-verbal signals, like facial expression, eye gaze, and posture, are a rich source of interpersonal communication signals. Reduced visual attention to these cues has been found in disorders of social development and some theories suggest a causal link between attending to facial communication cues and social deficits in autism spectrum disorder (ASD) (Campbell et al., 2006). The potential impact on social development of being born with no or very low levels of vision is therefore of high theoretical interest and clinical concern. Without visual interpersonal abilities, children with congenital visual impairment (VI) may be at higher risk of social deficits. They also provide a natural experiment to study the effects of absent vision from birth on the development of social cognition and behaviour. The current study, therefore, sets out to investigate if differences in social responses and behaviour, including social communicative/ASD risks, are found between children with congenital VI and typically-sighted controls, with a particular focus on neural responses toauditory social stimuli and their associations with parent-reported measures of social communicative and behavioural function and risk.

Children with congenital VI have been found to deviate from the typical trajectory of social development observed in typically-sighted children. Firstly, children with VI pass social cognitive Theory of Mind tests at a later age compared to typically-sighted children (Brambring and Asbrock, 2010, Peterson et al., 2000, Minter et al., 1998, Green et al., 2004, McAlpine and Moore, 1995). Secondly, a relatively large proportion of children with VI display behaviours that are commonly associated with autism, like stereotyped movement, echolalic speech, and lack of engagement with caregivers and peers (Preisler, 1991, Brown et al., 1997, Hobson and Bishop, 2003, Tadic et al., 2009). Difficulties in social processing persist in mid-childhood and potentially into adolescence. For instance, a study in a specialist secondary school indicated that 22% of students with VI met clinical criteria for ASD (Mukkades et al., 2007, also see Jure et al., 2016). Analyses of clinically referred samples suggest an even higher prevalence (Absoud et al., 2010, Williams et al., 2013, Rogers and Newhart-Larson, 2008). However, Hobson et al. (2010) provided some evidence of later ‘recovery’ from early symptomatology This potential change in the trajectory of social processing makes mid-childhood a particularly interesting period to investigate if qualitative differences in the processing of social stimuli can be detected.

Behavioural studies of social function in VI to date have some significant methodological limitations. First, many previous studies included congenital visual disorders that also involve central brain structures or included children with low intellectual functioning. It is therefore not clear if VI, low intellectual ability, or brain damage is affecting social development. In this study, we addressed these limitations by investigating social development in VI in a group of children with relatively pure disorders of the anterior visual system, who also scored in the normal range for verbal IQ.

Another potential limitation of previous studies concerns the behavioural assessment methods. Standard assessments of social cognition and Theory of Mind for sighted children rely predominantly on visually presented materials, e.g. recognising facial expressions or inferring the mental state of actors or puppets. Some attempts have been made to adapt materials for children with VI (Brambring and Asbrock, 2010), but these assessments have not been sufficiently tested and standardised in a representative sample − probably due to the rarity of isolated VI disorders. Other studies rely on verbally presented materials (Pijnacker et al., 2012) that are potentially confounded by differences in language development in children with VI, such as linguistic delays and difficulties in semantic and pragmatic comprehension (Andersen et al., 1993, Mulford, 1988, Wakefield et al., 2006). Therefore, the current study used a more direct measurement of auditory social stimulus processing in an electrophysiological experiment, which was not dependent on vision or language ability or verbal working memory.

We hypothesised that differences in the processing of social stimuli in children with VI would be apparent in responses to auditory social stimuli. The ‘subjects-own-name’ (SON) paradigm was considered particularly suitable as it assesses responses to a basic social stimulus in the auditory domain, ie. hearing one’s own name spoken. Own-name stimuli have been shown to be processed preferentially, automatically, and without conscious control in the typically-sighted child from as early as 5 months of age (Alexopoulos et al., 2012, Parise et al., 2010, Perrin et al., 2006, Pfister et al., 2012). Differences in processing of own-name stimuli, compared with typically developing children, have been associated with deficits in mentalizing ability in children with high-functioning autism (Cygan et al., 2014, Lombardo et al., 2009). In the event-related response, own-name stimuli consistently elicit an early negative deflection with a maximum over frontal channels followed by later positive deflection similar to the orienting-P3 (P3a) in typical adults (Tateuchi et al., 2012, Tateuchi et al., 2015). Manipulations of presentation frequency indicate that the early SON-related negativity is distinct from the orienting P3 (Eichenlaub et al., 2012). Further, comparisons of responses to names of close family members indicate that the SON-related negativity is specific to the subject’s own name rather than personally familiar names in general (Tateuchi et al., 2015). Neural generators of SON-related processing are likely to involve a network of areas, including the right superior temporal sulcus (STS), medial prefrontal cortex (PFC), and inferior parietal sulcus (IPS) (Holeckova et al., 2006, Perrin et al., 2005, Kampe et al., 2003). Using the event-related response in the SON paradigm, we anticipated that there may be a reduction in amplitude of the SON-related response in children with VI similar to effects observed in other groups with suspected deficits in social processing (Cygan et al., 2014, Nadig et al., 2007).

A school-aged sample of children with congenital disorders of the peripheral visual system (CDPVS), aged 8–13 years of normal verbal intelligence, were recruited for the investigation with matched controls of typical sight. Parent-reported questionnaires that are validated on children with typical sight for social communication difficulties, pragmatic language disorder, autism risk, and behavioural strengths and difficulties were included to assess the level of difficulties in the social domain. It was hypothesised further that individual differences between children with VI on the questionnaire measures (Sonksen and Dale, 2002, Tadic et al., 2009) would be associated with differences in the ERP response to SON.

## 2 Materials and methods

### 2.1 Participants

The assessments presented here were part of a wider study on the neural and cognitive sequelae of congenital VI during mid-childhood. This study was performed in accordance with the Declaration of Helsinki. The study was approved by Bloomsbury Research Ethics Committee − approval: 12/LO/0939. All parents, guardians or next of kin provided written informed consent and children provided verbal assent to participate in this study.

A prospective cross-sectional study was undertaken with eighteen children with VI aged between 8 and 13 years. Congenital disorders of the peripheral visual system with severe VI are rare with an estimated prevalence of less than 2–3 per 10,000 children (UK) raising challenges for recruitment and sampling (Rahi, Cable, BCVISG, 2003). Children were therefore recruited through national specialist clinics at Great Ormond Street Hospital for Children NHS Foundation Trust and Moorfields Eye Hospital NHS Foundation Trust. Inclusion criteria: i) children with congenital VI in the moderate to profound range with primary diagnosis of ‘potentially simple’ congenital disorders of the peripheral system (CDPVS), that is disorders affecting the globe of the eye, retina, or anterior optic nerve up to the optic chiasm, with no known brain disorder indicated by the paediatric or ophthalmological diagnosis (Sonksen and Dale, 2002), ii) between 8 and 13 years, iii) good verbal functioning (verbal IQ at the last assessment >75 or attending mainstream school at age-appropriate level), iv) English as their first language. Children with indications of additional neurological or endocrine abnormalities in their clinical records were excluded. Recruitment was undertaken through initial identification through clinical databases of children who had attended a tertiary paediatric specialist clinic at the hospital research site and open recruitment call through voluntary agencies associated with VI.

Children in the typically-sighted control group were recruited to match the same age range and were included if they fit the following criteria: attend mainstream school at the age-appropriate level, have no known neurological or psychiatric conditions, have either normal or corrected-to-normal vision, and have English as a first language. Sample characteristics are presented in Table 1, Table 2.

**Table 1:** Table Characteristics of participants in the VI group.

ID	Gender	Age [y]	VerbComp	logMAR	Near Detection	Vision Group	Visual Disorder	ERP
MVI 1	female	9.19	114	0.1	–	MVI	congenital nystagmus	✓
MVI 2	female	13.32	95	0.4	–	MVI	ocular fibrosis	✓
MVI 3	female	11.91	104	0.5	–	MVI	bilateral optic nerve hypoplasia	✓
MVI 4	male	12.34	–	0.54	–	MVI	rod-cone dystrophy	
MVI 5	female	8.27	104	0.6	–	MVI	oculocutaneous albinisim	✓
MVI 6	male	12.06	104	0.6	–	MVI	congenital nystagmus	✓
MVI 7	male	10.64	116	0.6	–	MVI	congenital nystagmus	✓
MVI 8	male	9.82	93	0.7	–	MVI	ocular albinism, congenital nystagmus	✓
MVI 9	female	12.26	96	left: 0.23, right: light perception	–	MVI, PVI	unilateral optic nerve hypoplasia	✓
SVI 1	female	10.98	87	0.9	–	SVI	hereditary progressive cone dystrophy	
SVI 2	male	11.69	148	0.9	–	SVI	oculocutaneous albinisim	✓
SVI 3	female	10.98	78	1.1	–	SVI	FEVR	
SVI 4	male	9.57	119	1.2	–	SVI	Leber’s congenital amaurosis	
SVI 5	male	9.01	–	1.225	–	SVI	ocular albinism, nystagmus	
SVI 6	male	9.91	96	1.225	–	SVI	Norrie’s disease	✓
SVI 7	female	11.04	75	–	1.5 cm sweet from 20 cm	SVI	Leber’s congenital amaurosis	
SVI 8	female	9.86	95	–	12.5 cm woolly from 50 cm	SVI	bilateral micro-ophthalmia	✓
PVI 1	male	10.36	134	–	light perception only	PVI	Leber’s congenital amaurosis	✓
	9 male	mean = 10.73	mean = 103.63					
	9 female	SE = 0.31	SE = 4.41					

Abbreviations: MVI: mild-to-moderate visual impairment, SVI: severe visual impairment, PVI: profound visual impairment, VerbComp: WISC-IV Verbal Comprehension age-normed score, FEVR: familial exudative vitreoretinopathy.

**Table 2:** Characteristics of participants in the typically-sighted control sample.

ID	Gender	Age [y]	VerbComp	logMAR	ERP
C1	female	8.56	98	−0.3	✓
C2	female	8.73	110	0.1	
C3	male	8.9	116	−0.3	
C4	male	9.08	102	0.1	✓
C5	female	9.12	98	−0.1	✓
C6	male	9.34	108	−0.2	
C7	male	10.07	96	0.1	✓
C8	male	10.16	134	0.0	✓
C9	male	10.37	106	0.0	✓
C10	male	10.74	102	−0.2	✓
C11	female	10.78	134	0.1	✓
C12	female	10.82	116	−0.2	✓
C13	female	10.89	83	0.0	
C14	female	11.09	130	−0.3	✓
C15	female	11.78	144	0.1	
C16	male	12.7	106	−0.2	✓
C17	male	12.77	130	−0.2	
C18	male	12.92	124	−0.3	
	8 female	mean = 10.49	mean = 113.17		
	10 male	SE = 0.32	SE = 3.87		

The full sample consisted of 18 children with VI (9 female) between 8 and 13 years of age (mean age: 10.76, age SD: 1.39, age range: 8.27-13.32) and a control group of 18 typically-sighted children (8 female, mean age: 10.62, age SD: 1.44, age range: 8.73-12.92). Verbal comprehension was assessed using verbal subtests of the Wechsler Intelligence Scale for Children 4th edition (WISC-IV) (Wechsler, 2004). Verbal subtests of previous and current editions of the WISC have also been used with children with VI (Greenaway et al., 2016, Dekker, 1993, Tillman, 1973, Tillman and Bashaw, 1968, Witkin et al., 1968). The administered subtests included all items of the Verbal Comprehension composite score (Vocabulary, Similarities, Comprehension). Two items were altered that required direct visual experience: The WISC-IV first practice item on the Similarities subtest which includes colour was not administered. The Comprehension question that asks about a situation in which ‘you see thick smoke’ was changed to ‘you smell thick smoke’. These alterations were used for the whole sample, including the typically-sighted control group. All other items were administered verbatim according to the WISC-IV administration manual (Wechsler, 2004). There was no significant difference in verbal IQ composite scores between the VI and the typically-sighted control group (VI: mean = 101.94, SE = 4.81, Range = 75-148; control: mean = 113.17, SE = 3.87, Range = 83-144; t(31.125) = −1.8193, p = 0.079).

Twenty-six children (72%) agreed to participate in the ERP part of the study (VI: 13; control: 13). One participant with VI was not included in the analysis because of excessive movement artefacts. One participant in the control group was rejected because of time-locked blinks that contaminated the ERP. One further participant in the control group was rejected because less than 10 trials were left after threshold artefact rejection (Final sample: VI = 12, control = 11). Complete parental questionnaires were available for all children with useable ERP data.

### 2.2 Assessment of vision level

The experimenter (J.B.) was trained by a neurodisability pediatrician specialised in VI to undertake the visual acuity assessments using the Sonksen logMAR test of Visual Acuity (Sonksen et al., 2008). For children who were not able to see the largest items on the Sonksen logMAR test, the Near Detection Scale was used to assess their basic level of detection vision (Sonksen et al., 1991).

Severe/Profound VI (S/PVI) is defined as limited form vision with logMAR above 0.8 (Snellen worse than 6/36) to no or light perception only (Near Detection Scale). Mild/moderate VI (MVI) is defined as reduced visual acuity with logMAR between 0.6 and 0.8 (Snellen 6/24-6/36).

### 2.3 ERP experiment

#### 2.3.1 Stimuli and stimulus presentation

The participants’ first name and a control name were recorded from 2 female and 2 male speakers in a quiet environment using Audacity software V 2.0.5 (http://audacity.sourceforge.net). The control name was selected to be unfamiliar, have the same number of syllables as the participant’s own name, and start with a different phoneme. In order to signal communicative intent, the recordings included “Hey” followed by the name (Kampe et al., 2003). Participants listened to a total of 80 stimuli in random order with balanced frequency for own-name and control-name stimuli. The experimental paradigms were implemented in MATLAB R2012b (The MathWorks, MA) using Psychtoolbox V3 functions (http://psychtoolbox.org).

The sound amplitude was normalised to 65 dB SPL for each stimulus in Audacity. Stimuli were presented through Creative Labs EP-660 in-ear headphones (Creative Labs Inc., Singapore). The headphones were electrically shielded by the manufacturer. Our own tests did not indicate that the headphones induced electrical artefacts above background noise in empty room recordings. Sound stimuli were presented through a Creative Sound Blaster X-Fi PCI Express sound card with a low latency driver (Creative Labs Inc., Singapore). Offset latency in this setup was measured and ERPs latencies were corrected accordingly.

#### 2.3.2 EEG recording

The EEG was recorded in a quiet darkened room using a GES 200 high-density, high-impedance recording system with a NetAmps 200 amplifier and HydroCel Geodesic Sensor Nets with 128 channels and suitable net sizes for all participants (Electrical Geodesics Inc., OR). Recordings were obtained using NetStation software V4.1.2. The EEG was recorded with a hardware low-pass filter at 400 Hz and a hard-ware high-pass filter at 0.01 Hz. The sampling frequency was set to 250 Hz. A vertex reference was used for recording.

#### 2.3.3 EEG processing for event-related potentials analysis

The EEG recordings were exported to EEGLAB format for processing and analysis. The pre-processing pipeline was based on previously described routines (Bathelt et al., 2014). First, the EEG was digitally filtered with finite impulse response (FIR) filters at a high-pass frequency of 0.1 Hz and a low-pass frequency of 30 Hz in EEGLAB 11.0.3 (Delorme and Makeig, 2004). Channels with low correlation to surrounding channels were rejected and interpolated from surrounding channels. The recording was re-referenced to the average reference. Second, the continuous EEG signal was segmented according to stimulus codes set during the recording beginning 100 ms before the onset of the recording to 500 ms post onset. Third, trials with absolute amplitudes higher than 150 μV in several channels were rejected. Baseline correction with the average amplitude in the 100 ms before the stimulus onset was applied. All epochs were visually inspected and epochs containing eye blinks, lateral eye movement, or movement artefact were rejected from further analysis.

There was no significant difference in the number of trials between participant groups (Independent two-sided *t*-test: VI: mean = 28.65, SE = 0.47, Range = 20-52; typically-sighted control: mean = 28.06, SE = 0.37, Range = 22-36, t(21) = 0.999, *p* = 0.32). There was a trend-level difference in the number of trials per conditions with fewer epochs for the control name (Independent two-sided *t*-test: Own name: mean = 29, SE = 0.57, Range = 20-52; control name: mean = 27.82, SE = 0.28, Range = 21-32; t(21) = 1.8682, *p* = 0.063). However, the difference in epochs was negligible with only 1 trial difference.

Channel groups and time windows of interest were selected based on previous reports using a similar paradigm (Holler et al., 2011a, Holler et al., 2011b). The channel areas of interest contained left and right frontolateral, left and right frontal, mid-frontal, and central channels (Channel labels according to the manufacturer: left frontolateral: 26, 27, 33, 34; right frontolateral: 3, 116, 122, 123; left frontal: 20, 24, 28, 29; right frontal: 111, 116, 117, 124; mid-frontal: 5, 6, 11, 12, central: 7, 31, 55, 80, 106). Channels within each area of interest were averaged to create a virtual channel. Three time windows were analysed: 50–150 ms after stimulus onset (N1 time window), 150–250 ms (P2 time window), and 280–320 ms (own name-related negativity). The N1 minimum over fronto-central channels appeared earlier in the VI group and fell outside the N1 range reported in the studies of typical adults (Holler et al., 2011). We therefore used an extended N1 window containing earlier latencies (50–150 ms). The mean amplitude in each time window was used for statistical comparison.

In addition to comparison of mean amplitude, an exploratory analysis of latency differences was carried out to investigate potential differences in the speed of processing between groups. The 50% fractional area latency was used as a robust measure of ERP latency (Kiesel et al., 2008).

### 2.4 Questionnaire measures of social communication, language use, and areas of difficulty

The levels of social communication, autism-related behaviours, and pragmatic language use were assessed using parent-reported questionnaires. The Social Communication Questionnaire (SCQ) focuses on the frequency of behaviours that are rare in typically developing children but common in children with an autism spectrum disorder (Rutter and Bailey, 2007). Standard clinical cut-offs were used to describe the results of this questionnaire. At the reported cut-offs, the specificity of distinguishing an ASD from other diagnoses is 0.96 (diagnostic range) and 0.80 (borderline range) (*Social Communication Questionnaire*, 2003).

The Children’s Communication Checklist 2nd edition (CCC) assesses pragmatic language use relative to structural language abilities (Bishop, 1998, Bishop, 2003). In addition, the Strengths and Difficulties questionnaire (SDQ) was administered to assess difficulties in areas of emotional symptoms, conduct problems, hyperactivity/inattention, peer problems, and prosocial behaviour (Goodman, 1997, Goodman et al., 2003).

All testing was carried out in a dedicated paediatric research facility. The questionnaires were filled in by a primary caregiver in a quiet waiting room, while the children participated in the ERP experiment.

The questionnaires were examined for references to visual behaviour prior to distribution. Five out of 40 items on the SCQ are indirectly related to vision. For instance, item 26 asks if the child looks at a person when talking to them. Other items may be vision dependent and include non-verbal gestures (e.g. item 24 “Does he/she nod his/her head to indicate no?"). Only one out of 70 items on the CCC-2 mentions vision-mediated behaviour, i.e. ‘Item 65: Smiles appropriately when talking to people’. We decided to include these items in the final analysis as the behaviour may be relevant for children with mild to moderate VI and to conserve the psychometric integrity of the questionnaire. During analysis, scoring the questionnaires including or pro-rating these items did not markedly influence the results of the study, i.e. statistically significant differences between the VI and typically-sighted group were found for both original and pro-rated scores.

Some questionnaire responses had to be excluded from the analysis because parents missed out items or decided not to fill in a questionnaire. For the SCQ, 13 parents in the VI group and 17 parents in the control group provided useable questionnaire responses. For the CCC, 17 parents in the VI group and 16 parents in the control group completed the questionnaire. For the SDQ, useable responses were available for 16 children in the VI group and 17 in the control group.

### 2.5 Statistical analysis

The age-related representative norms of the questionnaires were used to derive standard scores for analysis purposes. For the analysis of event-related potentials data, a repeated measures analysis of variance (ANOVA) model was used with factors for the channel region (left/right frontal, left/right fronto-lateral, mid-frontral, central), condition (own name, control name), and participant group (VI, control). The different time windows of interest (50–150 ms, 150–250 ms, 280–320 ms) were analysed seperately. Shapiro-Wilk tests on mean amplitudes for each channel region showed that normality assumptions were met. Mauchly’s test indicated that sphericity assumptions were not violated; therefore Greenhouse-Geisser correction was not necessary. Analysis of global effects was followed-up with Student *t*-tests corrected for multiple comparisons using Bonferroni correction. All statistical analyses were carried out in R version 3.2.3 (R Core Team, 2015). Visualisations were created using Matplotlib version 1.4.3 (Hunter, 2007) under IPython version 4.2.0 (Perez and Granger, 2007).

## 3 Results

### 3.1 ERP responses to SON stimuli in VI children compared with typically-sighted controls

**Fig. 1:**
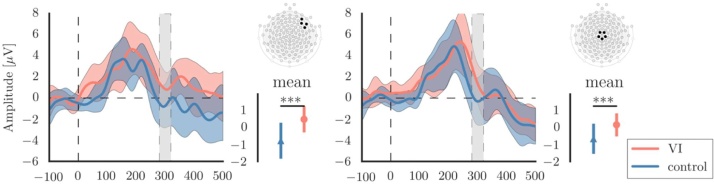
Grand-average ERP waveforms in the VI and control group for the own name condition for right frontal (left) and mid-central (right) channels. The solid line indicates the mean response, shaded areas indicate two standard errors around the mean. The grey boxes mark the time windows of interest (N280-320). The mean amplitude between the groups is shown next to the waveform. Statistical analysis indicated significantly higher mean amplitude in the VI group compared to typically-sighted controls in the 280–320 ms time window over right frontal and mid-central channels. The waveforms were low-pass filtered at 15 Hz for visualisation purposes.

The observed ERP waveforms displayed the expected morphology of components with a small early negative deflection followed by a large positive deflection over central and frontal channels in both VI and typically-sighted controls (see Fig. 1). A negative going wave between 270 and 320 ms after stimulus onset with larger amplitude in the own name condition over fronto-central channels was also observed (SON-related negativity) (Tateuchi et al., 2012).

The mean amplitudes in time windows corresponding to the N1 (50–150 ms), P2 (150–250), and SON-related negativity (280–320 ms) were analysed in a repeated-measures analysis of variance (rmANOVA) model with factors of condition, region, and participant group. The statistical analysis indicated an effect of condition in the earliest time window (50–150 ms) with more negative amplitudes in the own name condition (rmANOVA: Own name: mean = 0.69, SE = 0.16, Range = −6.21-4.26; Control name: mean = 1.47, SE = 0.21, Range = −4.88-8.06, F(1,209) = 11.05, p = 0.011). There was also a significant effect of channel region with more positive amplitudes in frontal channels (left fronto-lateral: mean = 0.98, SE = 0.25; right fronto-lateral: mean = 1.53, SE = 0.43; left frontal: mean = 0.98, SE = 0.25; right frontal: mean = 1.53, SE = 0.33; mid-frontal: mean = 1.67, SE = 0.34; central: mean = 0.71, SE = 0.26, F(5,209) = 10.647, p = 0.0013, Bonferroni-corrected p = 0.0078). Significant effects of region were also found in the second time window (150–250 ms) with the highest amplitudes over central channels (left fronto-lateral: mean = 0.2, SE = 0.42; right fronto-lateral: mean = 1.88, SE = 0.47; left frontal: mean = 1.93, SE = 0.37; right frontal: mean = 2.79, SE = 0.35; mid-frontal: mean = 3.56, SE = 0.45; central: mean = 2.65, SE = 0.35, F(5,209) = 10.266, p < 0.001, Bonferroni-corrected p = 0.0054). In summary, early time windows showed effects of condition and channel regions, but there was no significant difference between children with VI and typically-sighted controls.

A significant participant-group-by-condition-by-channel-region interaction was observed in the 270–320 ms time window (F(5,209) = 4.064, p = 0.002). Follow-up simple contrasts revealed significant differences between the groups with less negative amplitudes in the VI group over right frontal channels in the own name condition (Right frontal: VI: mean = 2.78, SE = 0.73, Range = −7.87-8.25; control: mean = −0.77, SE = 0.32, Range = −3.81 2.23; t(31.024) = 4.4525, *p <* 0.001; Central: VI: mean = 2.78, SE = 0.73; control: mean = −0.77, SE = 0.32; t(31.02) = 4.453, *p <* 0.001, Bonferroni-corrected *p* = 0.003, See Fig. 1).

An exploratory analysis of 50% fractional area latency was carried out to investigate potential differences in the speed of processing between groups. The analysis employed the same statistical approach as the main analysis of ERP mean amplitudes. No significant group effects were detected in the 50–150 ms (N1) and 150–250 (P2) time windows. The results indicated a significant group-by-region interaction with a shorter ERP latency in the control group for left frontal channels in the 280–320 ms (SON) time window (VI: mean = 210.5, SE = 2.08; control: mean = 197.33, SE = 2.27; t(37.79) = 4.2812, p < 0.001, Bonferroni-corrected *p* = 0.004). All other effects did not reach significance criteria (Bonferroni-corrected *p *> 0.05). However, left frontal channel in the control group showed little discernible event-related response with amplitudes around baseline variation. Because of the low area under the curve, the cumulative sum reaches 50% faster in channels with little activity. The waveform morphology indicated that responses in the VI and control group over left frontal channels are not comparable. The latency difference is therefore likely to reflect difference in response topography rather than speed of processing.

### 3.2 Comparison of parent-reported social communicative behaviours between the VI group and typically-sighted group

Social Communication Questionnaire: two participants with VI reached scores above the clinical cut-off according to the SCQ manual. One further participant with VI reached a score in the elevated range according to the questionnaire manual. The mean score in the VI group was higher compared to the control typically-sighted group (VI: mean = 9.15, Range:2–23; control: mean = 1.28, Range = 0–5), but below the elevated range (see Table 3). When removing vision-related SCQ items, 1 participant reached a score above the clinical cut-off, and 2 participants reached a score in the elevated range.

**Table 3:** Ratings of Social Communication Questionnaire (SCQ) in the VI and control group. Results for scores for an ASD group from another study are shown as a reference (data reproduced from Sasson et al. (2012)).

	VI		control		ASD^a^	
Domain	mean	SD	mean	SD	mean	SD
Total	9.15	7.30	1.28	1.41	22.32	6.47
Reciprocal Social Interaction	2.00	2.37	0.44	0.78	7.98	3.48
Communication	3.92	2.35	0.78	0.88	6.87	2.53
Restricted, Repetitive, and Stereotyped Patterns of Behaviour	3.00	3.02	0.33	1.41	5.88	1.86

a Data reproduced from Sasson et al. (2012) J. Neurodevelopmental Disorders.

**Fig. 2:**
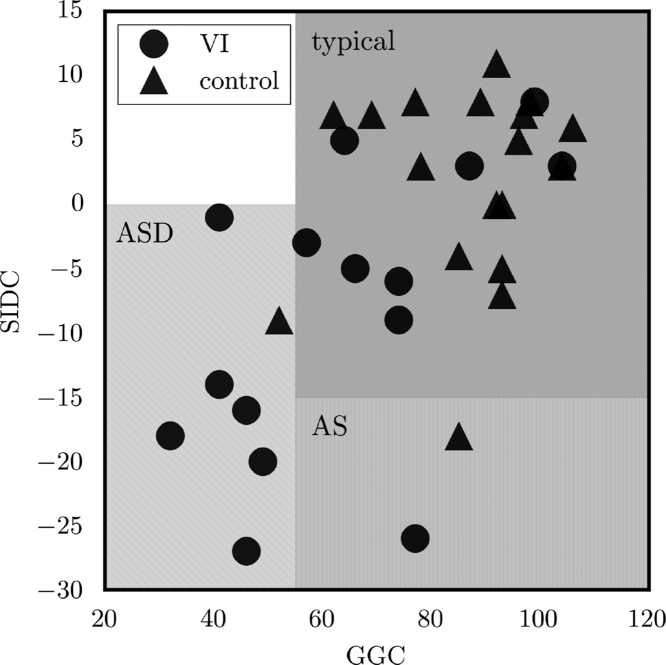
Distribution of General Communication Composite (GCC) and the Social Interaction Deviance Composite (SIDC) of the Children’s Communication Checklist 2nd edition (CCC) with reference to cut-off that warrant further clinical investigation of pragmatic language deficits frequently observed in children with either Asperger Syndrome (AS) or autism spectrum disorder (ASD). Cut-offs are defined as: ASD: GCC < 55, SIDC < 0; AS: GGC > 55, SIDC < −15 (Bishop and Norbury, 2002).

Children’s Communication Questionnaire (CCC 2nd edition): 4 children in the VI group reached Global Communication Composites below the 5%ile. Four children in the VI group and one child in the control group reached Social Deviance Composite Scores (SIDC) in the clinically significant range (Range: −16 to −27, see Fig. 2). The number of children reaching cut-off criteria on the Global Communication Composite or the Social Deviance Composite Score did not change when excluding the vision-related item.

**Fig. 3:**
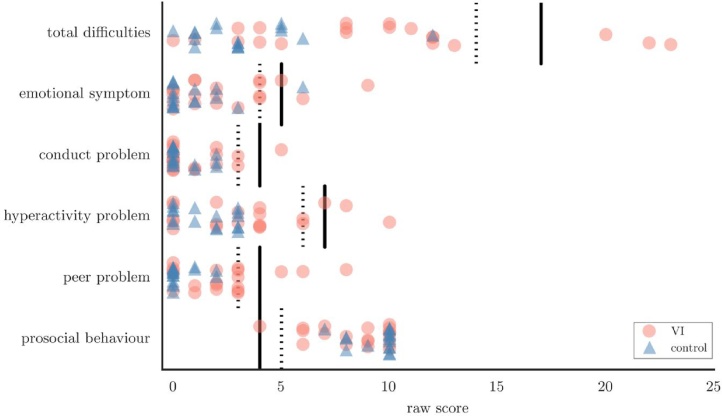
Ratings on the Strengths and Difficulties Questionnaire (SDQ). Participants in the VI group are shown as red circles and participants in the typically-sighted control group as blue triangles. The solid lines indicate the cut-off for abnormal scores according to the questionnaire manual. Dotted lines show the cut-off for the borderline range. Higher scores indicate more problems in each domain, apart from the prosocial behaviour scale where higher scores indicate better prosocial behaviour.

The Strengths and Difficulties Questionnaire (SDQ): Ratings on the SDQ indicated a high prevalence of everyday problems in the current sample of children with VI (see Fig. 3). Emotional problems and problems with peer relationships were most prevalent.

### 3.3 Relationship between SON ERP response and social communication questionnaire results

Bivariate correlations between mean amplitude between 280 and 320 ms in the own-name condition in the VI group and the CCC General Communication Composite, CCC Social Interaction Deviance Score, SDQ Peer Problems, and SDQ Emotional Problems were evaluated. A significant effect was found for SDQ Peer Problems that suggested fewer peer problems with more negative amplitudes (*n* = 12,  = −0.59, *p* = 0.044). However, the 95% probability level of the result did not survive stringent correction for multiple comparisons despite the large effect size (Bonferroni-corrected *p* = 0.176).

## 4 Discussion

The current study is the first to report neural correlates of social processing in children with visual impairment (VI). The auditory event-related potentials (ERP) evidence suggested that the sample of school-aged children with VI processed basic auditory social stimuli, i.e. recordings of their own name, differently compared to typically-sighted peers. The children with VI in the sample also showed some raised social communicative risk with elevated parental ratings of ASD-related behaviours, pragmatic language difficulties, and emotional and peer relationship problems. There was further tentative evidence that differences in neural processing of the auditory social stimuli related to greater behavioural difficulties with peers relationships in the VI group.

In a first step, the current study investigated the neurophysiological response to basic auditory social stimuli in children with VI in order to elucidate any differences in the neural processing of social signals. Differences between the own-name and control condition in the N1 and P2 time window indicated that own-name stimuli were preferentially processed. Higher amplitudes to self-relevant stimuli in early time windows are generally interpreted to reflect automatic capture of attention that does not require conscious awareness of the stimuli (Perrin et al., 1999; Perrin et al., 2006). Similar responses in both children with VI and typically sighted controls suggests that automatic attentional processing of self-relevant stimuli was not affected by congenital VI. In contrast to the current findings that indicated no differences in latency or amplitude of early ERP components, some studies reported enhanced auditory processing in congenitally blind adults characterised by larger amplitudes and early latencies of N1 (Roder et al., 1999, Roder et al., 2000). Differences in early-latency auditory processing in children with VI may only emerge later in development, may only be present in individuals with the most severe forms of VI, or may be more apparent with non-social stimuli. The current study used complex auditory stimuli and a low number of trails, which is not optimal to resolve subtle differences in early ERP components. Differences in auditory processing in children with VI should be addressed in future experiments.

While no differences in early components related to auditory processing and responses to control names between children with VI and typically-sighted controls were detected, group differences emerged in a later time window with less negative amplitudes to own-name stimuli in the VI group compared to controls. Similarly reduced amplitude of the ERP in response to own-name stimuli has also been reported for adults with ASD (Cygan et al., 2014). Responses to own name stimuli as presented in this study are thought to engage more social cognitive processing such as Theory of Mind (ToM) processing. Differences in the responsiveness to own name stimuli have been associated with deficits in Theory of Mind in ASD across several paradigms (Cygan et al., 2014, Lombardo et al., 2009Carmody and Lewis, 2011Cygan et al., 2014, Lombardo et al., 2009; Carmody and Lewis, 2011). Reduced behavioural responsiveness to self-related information, e.g lack of response to name calling in situ, are now even part of clinical diagnostic observational schedules for autism e.g. the Autism Diagnostic Observation Schedule (ADOS, Lord et al., 2008). The current study found reduced amplitudes in response to own name stimuli over central and right frontal channels. Central channel regions also show the largest own-name specific effects in typically-sighted adults (Eichenlaub et al., 2012, Muller and Kutas, 1996). The topography of the SON response in the VI group deviates from the topography in the typically-sighted control group and from the published results for typical adults. This may indicate that different neural substrates are engaged in own-name processing in children with VI. This could be associated with deficits in social processing or may indicate compensatory engagement of additional neural substrates in the VI group.

In summary, while early ERP components related to early-latency auditory processing did not differ between the groups, a specific deflection to own name stimuli over central and right frontal channels at later latencies was found to be attenuated in children with VI compared to typically sighted controls potentially indicating that auditory social stimuli are processed differently in children with congenital VI.

In a second step, the current study aimed to assess the level of social cognitive and social communicative function in this sample of high-functioning children with congenital VI. As in other studies on children with VI, this study indicated that children with VI are at higher risk of social communicative problems and show an increase in behaviours associated with autism spectrum disorder (ASD) on a variety of parent-reported measures. 2 out of 17 (12%) met the clinical cut-off (score >15) for ASD and a total of 3 out of 17 (18%) scored above the elevated cut-off. Previous studies used a range of different behavioural measures so it is difficult to compare ‘incidence’ but this number appears lower than previously reported (Brown et al., 1997, Mukkades et al., 2007, Rogers and Newhart-Larson, 2008, Williams et al., 2013, Jutley-Neilson et al., 2013, Parr et al., 2010, Jure et al., 2016). However, in previous studies likelihood of meeting ASD criteria was associated with lower vision level and lower verbal ability (Brown et al., 1997). The current sample was selected for good verbal abilities to avoid confounding effects of additional learning disabilities. Further, half of the sample consisted of children with mild to moderate VI, while most previous studies focused on severe to profound VI. The lower incidence in this higher-functioning sample may suggest that better verbal ability and greater visual function may play a protective role as suggested by other authors (Brown et al., 1997) and that severity of vision level, particularly profound VI (light perception at best), is a risk factor for early social communicative difficulties and ASD (Absoud et al., 2010). Nonetheless, the 3 children who met ASD/AS criteria were neither the children with the lowest vision level nor the lowest verbal ability. This may indicate that other factors may be involved in aetiology or maintenance of these difficulties.

The results of the pragmatic language and strengths and difficulties questionnaire indicated that there were more widespread subtle difficulties or deficits in social function in this group of higher functioning children with VI (Harris and Lord, 2016) similar to previously reported findings in an independent sample with similar characteristics (Tadic et al., 2009). These difficulties included problems with peer relationships, deficits in emotional regulation, insufficient use of context in conversation, and stereotyped language among other difficulties. This may indicate that children with congenital VI show similarities to children that fall within the wider autism spectrum (Reisinger et al., 2011, Taylor et al., 2013). However, elevated scores have also been found for neurodevelopmental disorders like ADHD (Cooper et al., 2013), preterm birth (Johnson et al., 2010), and children with pragmatic language impairment (Reisinger et al., 2011), suggesting that milder tendencies towards the wider autistic spectrum or certain aspects of the autistic spectrum may be a common feature of atypical development that is also present in children with congenital VI.

The mechanisms leading to differences in social processing and increased risk of social deficits in congenital VI are currently not known. Following an interactive specialisation view (Johnson, 2011), by which differences in one function may have cascading effects on the development of other functions, we speculate that absent access to visual social cues, specifically eye gaze and facial expression, may play an important role in the aetiology of atypical social development in children with congenital VI (Campbell et al., 2006) mirroring some theories that have been proposed in the ASD literature (Klin et al., 2002). Eye gaze discrimination is believed to be key for the development of joint attention in infancy with important consequences for sociocognitive development (Moore and Dunham, 1994). Reciprocal interaction between mother and infant has been linked to the development of social communication and social cognition (Moore and Dunham, 1994).

At the neurophysiological level, differences in visual input or visual drive may have lead to altered development of the social processing network (Eggebrecht et al., 2017). Facial expressions are processed in a specific network, including the posterior superior temporal sulcus, ventromedial prefrontal cortex, and temporal pole (Leppanen and Nelson, 2008), which receives visual input from the fusiform face areas (FFA). The same areas also show higher blood oxygenation during sociocognitive processing (Frith and Frith, 2003, Kampe et al., 2003). Thus, both facial expressions and sociocognitive reasoning are at least in part processed in the same circuit. The current study shows an attenuated ERP in the children with VI using a paradigm (SON) that has been shown to engage the social processing circuit (Kampe et al., 2003). We propose that differences in the processing of auditory social stimuli in congenital VI may have arisen as a consequence of reduced or missing input from areas involved in the processing of visual social information, which lead to downstream effects on the development of the social processing network. This idea remains to be investigated in detail in future longitudinal studies employing additional neuroimaging modalities and additional comparison groups of typically-sighted children with atypical social development.

### 4.1 Limitations

The current study has some limitations which affect generalisability of findings. First, the sample size was very limited, which is mainly due to the population rarity of the congenital disorders of the peripheral visual system and consequent recruitment challenges (2–3 per 10,000 children in the UK, Rahi and Cable, 2003). An associated potential issue concerns the heterogeneity of the sample. In order to reach a sample size that allowed for a meaningful group comparison, a range of disorders was included that share common impairment, i.e. visual impairment and CNS involvement restricted to the peripheral visual system. The individual disorders are extremely rare with often little understood and complex genetic causes so that heterogeneity is even found within diagnostic categories. However, investigations with larger clinical samples found autistic features across the range of congenital disorders of the peripheral visual system (Dale and Sonksen, 2002, Sonksen and Dale, 2002, Absoud et al., 2010, Parr et al., 2010).

The representativeness of the current sample also demands cautious interpretation. Children were recruited to show an intellectual function within the typical range for their age, which is not the case for a large proportion of children with visual impairment who have additional intellectual disabilities (Alimovic, 2012, Rahi and Cable, 2003). Further, children were mostly recruited through specialised clinical services so that the sample is potentially clinically biased towards children with specific problems even though these children were mostly referred for standard clinical care during the early years and were later discharged.

A further limitation is that only parental questionnaire ratings of social communication and behavioural difficulties or ASD criteria were collected without further validation by behavioural measures or teacher-rated questionnaires. In the future, further investigation of social communicative ‘risk’ and behavioural difficulties will be improved by using robust observational assessments that are specifically designed for and validated on children with VI and that do not depend on vision behaviours.

The study is cross-sectional and therefore it is not possible to conclude if VI is playing a causal role and in the future longitudinal studies may provide more insight into underlying mechanisms.

### 4.2 Conclusion

The current study investigated if social difficulties and deficits in high-functioning children with congenital VI are associated with different processing of basic auditory social stimuli using the event-related potentials method. Results of the ERP investigation suggested differences in the response to social stimuli in the VI group compared with typically-sighted children, suggesting that VI may have a function in neural development underpinning the SON paradigm. The results also indicated that social and pragmatic deficits are common in the children with VI in line with previous behavioural studies. There was also tentative evidence that neural differences may relate to everyday difficulties with peer relationships. Notwithstanding limitations regarding sample size and generalizability, the findings provide proof-of-concept evidence suggesting that visual stimuli or visual function may play an important role in basic social processing (SON) that possibly has associations with other areas of social cognition.

Evidence of differences in the basic neural response to social stimuli highlights both clinical risk and also the importance of developing and evaluating potential intervention and habilitative avenues in the future.

## Conflict of Interest

None.

## References

[bib0005] Absoud M., Parr J.R., Salt A., Dale N. (2010). Developing a schedule to identify social communication difficulties and autism spectrum disorder in young children with visual impairment. Dev. Med. Child Neurol..

[bib0010] Alexopoulos T., Muller D., Ric F., Marendaz C. (2012). I me, mine: automatic attentional capture by self-related stimuli. Eur. J. Soc. Psychol..

[bib0015] Alimovic S. (2012). Emotional and behavioural problems in children with visual impairment intellectual and multiple disabilities. J. Intellect. Disabil. Res..

[bib0020] Andersen E.S., Dunlea A., Kekelis L. (1993). The impact of input: language acquisition in the visually impaired. First Lang..

[bib0025] Bathelt J., OReilly H., de Haan M. (2014). Cortical source analysis of high-density eeg recordings in children. J. Vis. Exp..

[bib0030] Bishop D.V., Norbury C.F. (2002). Exploring the borderlands of autistic disorder and specific language impairment: a study using standardised diagnostic instruments. J. Child Psychol. Psychiatry.

[bib0035] Bishop D.V.M. (1998). Development of the childrens communication checklist (CCC): a method for assessing qualitative aspects of communicative impairment in children. J. Child Psychol. Psychiatry.

[bib0040] Bishop D.V.M. (2003). The Children’s Communication Checklist.

[bib0045] Brambring M., Asbrock D. (2010). Validity of false belief tasks in blind children. J. Autism Dev. Disord..

[bib0050] Brown R., Hobson R.P., Lee A., Stevenson J. (1997). Are there autistic-like features in congenitally blind children?. J. Child Psychol. Psychiatry.

[bib0055] Campbell R., Lawrence K., Mandy W., Mitra C., Jeyakuma L., Skuse D. (2006). Meanings in motion and faces: developmental associations between the processing of intention from geometrical animations and gaze detection accuracy. Dev. Psychopathol..

[bib0060] Carmody D.P., Lewis M. (2011). Self representation in children with and without autism spectrum disorders. Child Psychiatry Hum. Dev..

[bib0065] Cooper M., Martin J., Langley K., Hamshere M., Thapar A. (2013). Autistic traits in children with ADHD index clinical and cognitive problems. Eur. Child Adolesc. Psychiatry.

[bib0070] Cygan H.B., Tacikowski P., Ostaszewski P., Chojnicka I., Nowicka A. (2014). Neural correlates of own name and own face detection in autism spectrum disorder. PLoS One.

[bib0075] Dale N., Sonksen P. (2002). Developmental outcome including setback, in young children with severe visual impairment. Dev. Med. Child Neurol..

[bib0080] Dekker R. (1993). Visually impaired children and haptic intelligence test scores: intelligence test for visually impaired children (ITVIC). Dev. Med. Child Neurol..

[bib0085] Delorme A., Makeig S. (2004). EEGLAB: an open source toolbox for analysis of single-trial EEG dynamics including independent component analysis. J. Neurosci. Methods.

[bib0090] Eggebrecht A.T., Elison J.T., Feczko E., Todorov A., Wolff J.J., Kandala S. (2017). Joint attention and brain functional connectivity in infants and toddlers. Cereb. Cortex.

[bib0095] Eichenlaub J.-B., Ruby P., Morlet D. (2012). What is the specificity of the response to the own first-name when presented as a novel in a passive oddball paradigm? An ERP study. Brain Res..

[bib0100] Frith U., Frith C.D. (2003). Development and neurophysiology of mentalizing. Phil. Trans. R. Soc. B : Biol. Sci..

[bib0105] Goodman R., Ford T., Simmons H., Gatward R., Meltzer H. (2003). Using the Strengths and Difficulties Questionnaire (SDQ) to screen for child psychiatric disorders in a community sample. Int. Rev. Psychiatry.

[bib0110] Goodman R. (1997). The strengths and difficulties questionnaire: a research note. J. Child Psychol. Psychiatry.

[bib0115] Green S., Pring L., Swettenham J. (2004). An investigation of first-order false belief understanding of children with congenital profound visual impairment. Br. J Dev. Psychol..

[bib0120] Greenaway R., Pring L., Schepers A., Isaacs D.P., Dale N.J. (2016). Neuropsychological presentation and adaptive skills in high-Functioning adolescents with visual impairment: a preliminary investigation. Appl. Neuropsychol.: Child.

[bib0125] Harris J., Lord C. (2016). Mental health of children with vision impairment at 11 years of age. Dev. Med. Child Neurol..

[bib0130] Hobson P.R., Bishop M. (2003). The pathogenesis of autism: insights from congenital blindness. Phil. Trans. R. Soc. B : Biol. Sci..

[bib0135] Hobson R.P., Lee A., Lee A. (2010). Reversible autism among congenitally blind children? A controlled follow-up study. J. Child Psychol. Psychiatry.

[bib0140] Holeckova I., Fischer C., Giard M.-H., Delpuech C., Morlet D. (2006). Brain responses to a subjects own name uttered by a familiar voice. Brain Res..

[bib0145] Holler Y., Kronbichler M., Bergmann J., Crone J.S., Ladurner G., Golaszewski S. (2011). EEG frequency analysis of responses to the own-name stimulus. Clin. Neurophysiol..

[bib0150] Holler Y., Kronbichler M., Bergmann J., Crone J.S., Schmid E.V., Golaszewski S., Ladurner G. (2011). Inter-individual variability of oscillatory responses to subjects own name. A single-subject analysis. Int. J. Psychophysiol..

[bib0155] Hunter J.D. (2007). Matplotlib: a 2D graphics environment. Comput. Sci. Eng..

[bib0160] Johnson M.H., Dziurawiec S., Ellis H., Morton J. (1991). Newborns preferential tracking of face-like stimuli and its subsequent decline. Cognition.

[bib0165] Johnson M.H., Farroni T., Brockbank M., Simion F. (2000). Preferential orienting to faces in 4-month-olds: analysis of temporal-nasal visual field differences. Dev. Sci..

[bib0170] Johnson S., Hollis C., Hennessy E., Kochhar P., Wolke D., Marlow N. (2010). Screening for autism in preterm children: diagnostic utility of the Social Communication Questionnaire. Arch. Dis. Child..

[bib0175] Johnson M.H. (2011). Interactive specialization: a domain-general framework for human functional brain development?. Dev. Cognit. Neurosci..

[bib0180] Jure R., Pogonza R., Rapin I. (2016). Autism Spectrum Disorders (ASD) in blind children: very high prevalence, potentially better outlook. J. Autism Dev. Disord..

[bib0185] Jutley-Neilson J., Harris G., Kirk J. (2013). The identification and measurement of autistic features in children with septo-optic dysplasia optic nerve hypoplasia and isolated hypopituitarism. Res. Dev. Disabil..

[bib0190] Kampe K.K.W., Frith C.D., Frith U. (2003). Hey John: signals conveying communicative intention toward the self activate brain regions associated with mentalizing, regardless of modality. J. Neurosci..

[bib0195] Kiesel A., Miller J., Jolicoeur P., Brisson B. (2008). Measurement of ERP latency differences: a comparison of single-participant and jackknife-based scoring methods. Psychophysiology.

[bib0200] Klin A., Jones W., Schultz R., Volkmar F., Cohen D. (2002). Visual fixation patterns during viewing of naturalistic social situations as predictors of social competence in individuals with autism. Arch. Gen. Psychiatry.

[bib0205] Leppanen J.M., Nelson C.A. (2008). Tuning the developing brain to social signals of emotions. Nat. Rev. Neurosci..

[bib0210] Lombardo M.V., Chakrabarti B., Bullmore E.T., Sadek S.A., Pasco G., Wheelwright S.J., Suckling J., Baron-Cohen S. (2009). Atypical neural self-representation in autism. Brain.

[bib0215] Lord C., Rutter M., DeLavore P.C., Risi S. (2008). Autism Diagnostic Observation Schedule.

[bib0220] McAlpine L.M., Moore C.L. (1995). The development of social understanding in children with visual impairments. J. Visual Impair. Blindness.

[bib0225] Minter M., Hobson R.P., Bishop M. (1998). Congenital visual impairment and ‘theory of mind’. Br. J Dev. Psychol..

[bib0230] Moore C., Dunham P.J. (1994). Joint Attention: Its Origins and Role in Development.

[bib0235] Mukkades N.M., Kilincaslan A., Kucukyazici G., Sevketoglu T., Tuncer S. (2007). Autism in visually impaired individuals. Psychiatry Clin. Neurosci..

[bib0240] Mulford R., Smith M.D., Locke J.L. (1988). First words of the blind child: the child’s development of a linguistic vocabulary. The Emergent Lexicon.

[bib0245] Muller H.M., Kutas M. (1996). What’s in a name? Electrophysiological differences between spoken nouns proper names and one’s own name. Neuroreport.

[bib0250] Nadig A.S., Ozonoff S., Young G.S., Rozga A., Sigman M., Rogers S.J. (2007). A prospective study of response to name in infants at risk for autism. Arch. Pediatr. Adolesc. Med..

[bib0255] Parise E., Friederici A.D., Striano T. (2010). Did You Call Me? 5-month-old infants own name guides their attention. PLoS One.

[bib0260] Parr J.R., Dale N.J., Shaffer L.M., Salt A.T. (2010). Social communication difficulties and autism spectrum disorder in young children with optic nerve hypoplasia and/or septo-optic dysplasia. Dev. Med. Child Neurol..

[bib0265] Perez F., Granger B.E. (2007). IPython: a system for interactive scientific computing. Comput. Sci. Eng..

[bib0270] Perrin F., Maquet P., Peigneux P., Ruby P., Degueldre C., Balteau E., Del Fiore G., Moonen G., Luxen A., Laureys S. (2005). Neural mechanisms involved in the detection of our first name: a combined ERPs and PET study. Neuropsychologia.

[bib0275] Perrin F., Schnakers C., Schabus M., Degueldre C., Goldman S., Bredart S., Faymonville M.-E., Lamy M., Moonen G., Luxen A., Maquet P., Laureys S. (2006). Brain response to ones own name in vegetative state minimally conscious state, and locked-in syndrome. Arch. Neurol..

[bib0280] Peterson C.C., Peterson J.L., Webb J. (2000). Factors influencing the development of a theory of mind in blind children. Br. J Dev. Psychol..

[bib0285] Pfister R., Pohl C., Kiesel A., Kunde W. (2012). Your unconscious knows your name. PLoS One.

[bib0290] Pijnacker J., Vervloed M.P.J., Steenbergen B. (2012). Pragmatic abilities in children with congenital visual impairment: an exploration of non-literal language and advanced theory of mind understanding. J. Autism Dev. Disord..

[bib0295] Preisler G.M. (1991). Early patterns of interaction between blind infants and their sighted mothers. Child: Care Health Dev..

[bib0300] R Core Team (2015). R: A Language and Environment for Statistical Computing.

[bib0305] Rahi J.S., Cable N. (2003). Severe visual impairment and blindness in children in the UK. Lancet.

[bib0310] Reisinger L.M., Cornish K.M., Fombonne E. (2011). Diagnostic differentiation of autism spectrum disorders and pragmatic language impairment. J. Autism Dev. Disord..

[bib0315] Roder B., Teder-Sälejärvi W., Sterr A., Rösler F., Hillyard S.A., Neville H.J. (1999). Improved auditory spatial tuning in blind humans. Nature.

[bib0320] Roder B., Rosler F., Neville H.J. (2000). Event-related potentials during auditory language processing in congenitally blind and sighted people. Neuropsychologia.

[bib0325] Rogers S.J., Newhart-Larson S. (2008). Characteristics of infantile autism in five cases of Leber’s congenital amaurosis. Dev. Med. Child Neurol..

[bib0330] Rutter M., Bailey A. (2007). The Social Communication Questionnaire.

[bib0335] Sasson N.J., Nowlin R.B., Pinkham A.E. (2012). Social cognition social skill, and the broad autism phenotype. Autism.

[bib0340] Sonksen P.M., Dale N. (2002). Visual impairment in infancy: impact on neurodevelopmental and neurobiological processes. Dev. Med. Child Neurol..

[bib0345] Sonksen P.M., Petrie A., Drew K.J. (1991). Promotion of visual development of severely visually impaired babies: evaluation of a developmentally based programme. Dev. Med. Child Neurol..

[bib0350] Sonksen P.M., Wade A.M., Proffitt R., Heavens S., Salt A.T. (2008). The Sonksen logMAR test of visual acuity: II. Age norms from 2 years 9 months to 8 years. J. AAPOS.

[bib0355] Tadic V., Pring L., Dale N. (2009). Are language and social communication intact in children with congenital visual impairment at school age?. J. Child Psychol. Psychiatry.

[bib0360] Tateuchi T., Itoh K., Nakada T. (2012). Neural mechanisms underlying the orienting response to subjects own name: an event-related potential study. Psychophysiology.

[bib0365] Tateuchi T., Itoh K., Nakada T. (2015). Further characterization of subject’s own name (SON) negativity, an ERP component reflecting early preattentive detection of SON. BMC Res. Notes.

[bib0370] Taylor L.J., Maybery M.T., Wray J., Ravine D., Hunt A., Whitehouse A.J.O. (2013). Brief Report: do the nature of communication impairments in autism spectrum disorders relate to the broader autism phenotype in parents?. J. Autism Dev. Disord..

[bib0375] Tillman M.H., Bashaw W.L. (1968). Multivariate analysis of the WISC scales for blind and sighted children. Psychol. Rep..

[bib0380] Tillman M.H. (1973). Intelligence scales for the blind: a review with implications for research. J. School Psychol..

[bib0385] Wakefield C.E., Homewood J., Taylor A.J. (2006). Early blindness may be associated with changes in performance on verbal fluency tasks. J. Vis. Impair. Blindness.

[bib0390] Wechsler D. (2004). The Wechsler Intelligence Scale for Children − Fourth UK Edition.

[bib0395] Williams M.E., Fink C., Zamora I., Borchert M. (2013). Autism assessment in children with optic nerve hypoplasia and other vision impairments. Dev. Med. Child Neurol..

[bib0400] Witkin H.A., Birnbaum J., Lomonaco S., Lehr S., Herman J.L. (1968). Cognitive patterning in congenitally totally blind children. Child Dev..

